# Effects of Vitamin D and Calcium Fortified Yogurts on Gait, Cognitive Performances, and Serum 25-Hydroxyvitamin D Concentrations in Older Community-Dwelling Females: Results from the GAit, MEmory, Dietary and Vitamin D (GAME-D2) Randomized Controlled Trial †

**DOI:** 10.3390/nu11122880

**Published:** 2019-11-26

**Authors:** Olivier Beauchet, Cyrille P. Launay, Kevin Galery, Christine Vilcocq, Flore Dontot-Payen, Brigitte Rousseau, Valérie Benoit, Gilles Allali

**Affiliations:** 1 Department of Medicine, Division of Geriatric Medicine, Sir Mortimer B. Davis-Jewish General Hospital and Lady Davis Institute for Medical Research, McGill University, Montreal, QC H3T 1E2, Canada; cyrille.launay@gmx.fr (C.P.L.); kevin.galery@mail.mcgill.ca (K.G.); christine.vilcocq@mail.mcgill.ca (C.V.); 2 Geriatric Medicine, Faculty of Medicine, McGill University, Montreal, QC H3T 1E2, Canada; 3 Centre of Excellence on Longevity of McGill Integrated University Health Network, Montreal, QC H3T 1E2, Canada; 4 Lee Kong Chian School of Medicine, Nanyang Technological University, Singapore 308232, Singapore; 5 Nutrition & Regulatory Affairs, Yoplait SAS, 92641 Boulogne-Billancourt, France; Flore.Dontot-Yoplait@genmills.com (F.D.-P.);; 6 Regulatory Affairs, Les Maîtres Laitiers du Cotentin, 50260 Sottevast, France; 7 The Bell Institute of Health and Nutrition, General Mills Inc., Minneapolis, MN 55427, USA; Valerie.Benoit@genmills.com; 8 Department of Neurology, Geneva University Hospital and University of Geneva, 1205 Genève, Switzerland; gilles.allali@hcuge.ch

**Keywords:** older adults, randomized controlled trial, hypovitaminosis D, gait, cognition

## Abstract

Background: Vitamin D_3_ fortified food may improve serum vitamin D level, suggesting that the prevention of adverse consequences of hypovitaminosis D is possible with food fortification. The aim of this randomized controlled trial (RCT) was to examine the effects of vitamin D and calcium fortified yogurt on spatiotemporal gait parameters, cognitive performance, handgrip strength, and serum 25OHD levels in healthy older females. Methods: Forty older community-dwelling females were recruited in a single-blind, randomized, controlled, superiority clinical trial in two parallel groups (20 participants in the intervention group and 20 in the control group) with intent-to-treat. The intervention group took fortified yogurts daily (i.e., 400 UI of vitamin D_3_ and 800 mg calcium) for 3 months. The non-fortified yogurts contained similar proteins, carbohydrates and lipids, as well as a lower dose of calcium (300 mg) and no vitamin D_3_ supplementation. Spatiotemporal gait parameters (mean value and coefficient of variation) were assessed using a computerized walkway. Handgrip strength was measured with hydraulic dynamometers. Cognitive performances, including global cognitive functioning assessed with the Mini Mental Status Examination (MMSE) were recorded. All the outcomes were assessed at baseline and at the end of follow-up. The primary outcome was the coefficient of variation of stride time. Results: The intervention group maintained its global cognitive performance and serum 25OHD concentrations, whereas these outcomes decreased (i.e., worst performance) in the control group. The changes in the MMSE score (*p* = 0.022) and serum 25OHD concentrations were different (*p* ≤ 0.001) with better values reported in the intervention group compared to the control group. There was no significant change in gait parameters (*p* ≥ 0.518) and handgrip strength (*p* ≥ 0.600). Conclusions: Fortified yogurts with vitamin D (i.e., 200 IU) and calcium (i.e., 400 mg) twice a day maintained global cognitive performance and vitamin D status in older females, but not gait performances, signifying that they mainly prevent hypovitaminosis D-related extra-skeletal disorders.

## 1. Introduction

Hypovitaminosis D is defined by low serum 25-hydroxyvitamin D (25OHD) concentrations [1,2]. A serum 25OHD concentration below 50 nmol/L (i.e., vitamin D deficiency) results in skeletal adverse consequences, including decreased bone mineralisation and secondary hyperparathyroidism [2]. Extra-skeletal adverse consequences, such as gait and cognitive impairments have also been reported with low serum 25OHD concentrations between 50 and 75 nmol/L (i.e., vitamin D insufficiency) [3,4,5]. Gait impairments are caused by physiological system impairments that depend, in part, on vitamin D-related metabolism [4,5,6,7]. Combined skeletal and extra-skeletal adverse consequences of hypovitaminosis D on the different subsystems involved in gait control, including the muscular and cognitive functions, may explain gait impairment [6,7]. In particular, increased stride time (i.e., gait cycle duration) variability, which is a sensitive and specific biomarker of abnormal gait control and gait instability, has been associated with hypovitaminosis D [6].

Hypovitaminosis D is highly prevalent in the older population and often affects up to 50% of elderly community dwellers [3,4]. Seasonal changes influence 25OHD serum concentrations, with a prevalence of hypovitaminosis D that may double in winter compared to the summer months [8,9]. Improving the vitamin D status of the older population to prevent hypovitaminosis D-related skeletal and extra-skeletal adverse consequences is recognized as an important public commitment [8,9,10]. Vitamin D supplementation and food fortification are two solutions to avoid seasonal fluctuations of serum 25OHD concentration [10,11,12,13,14,15,16]. There are two issues with vitamin D supplementation. Extremely high doses of vitamin D may expose individuals to adverse effects, such as an increased risk for falls and fractures, in addition to low compliance, which may limit its benefits [17]. Food fortification may be a good alternative to vitamin D supplementation [10]. In particular, yogurt fortified with vitamin D and calcium offers an appropriate solution [10,14,15]. A systematic review demonstrated that low daily doses (i.e., ≤800 IU) of vitamin D found in fortified food significantly increased serum 25OHD concentrations [16]. In addition, yogurt contains calcium—a key component for the mechanism of vitamin D and protein action—which helps to maintain healthy muscle mass [18,19].

To date, no randomized controlled trial (RCT) has examined changes in gait, cognitive and muscle performances in older females with hypovitaminosis D. We hypothesized that low daily doses of vitamin D (i.e., 400 IU) associated with 800 mg calcium through fortified yogurts during a 3-month period could maintain and/or improve gait, cognitive and muscle performances during fall and winter seasons. The study aimed to examine the effects of fortified yogurts with vitamin D and calcium on the variability of spatio-temporal gait parameters, especially on the coefficient of variation (CoV) of stride time used as the primary outcome, as well as on cognitive performances, handgrip strength and serum 25OHD levels in healthy older females.

## 2. Materials and Methods

### 2.1. Population 

This study was carried out in Angers and surrounding municipalities in France. Both urban and rural areas around Angers were included. Forty females were recruited between September 2014 and January 2015. The inclusion criteria were: female, age ≥ 65, living independently at home or in an autonomous residence, free of dementia, hypovitaminosis D (i.e., serum 25OHD concentration < 75 nmol/L), calcemia < 2.65 mmol/L, no vitamin D and/or calcium supplementation, oral and written comprehension of French and consent to participate. The exclusion criteria were contra-indications for vitamin D_3_ supplementation, history of calcium lithiasis, dairy allergies, severe hepatic and/or renal deficiency, lactose intolerance, exposure to sunlight in other countries than the country of recruitment or to artificial ultraviolet UVB during the 3-month participation period in the study, acute medical illness in the month preceding the baseline assessment, neurological diseases (i.e., Parkinson’s disease, cerebellar disease, myelopathy, and peripheral neuropathy), major orthopedic diagnoses (e.g., osteoarthritis) involving the lumber vertebra, pelvis or lower extremities, moderate depressive symptoms with a 15-item Geriatric Depression Scale (GDS) score ≥ 5/15 [20], inability to walk 15 meters unassisted, planned relocation outside of the recruitment living area during the follow-up period of the study, and participation (or commitment to participate) in another clinical trial.

**Figure 1:**
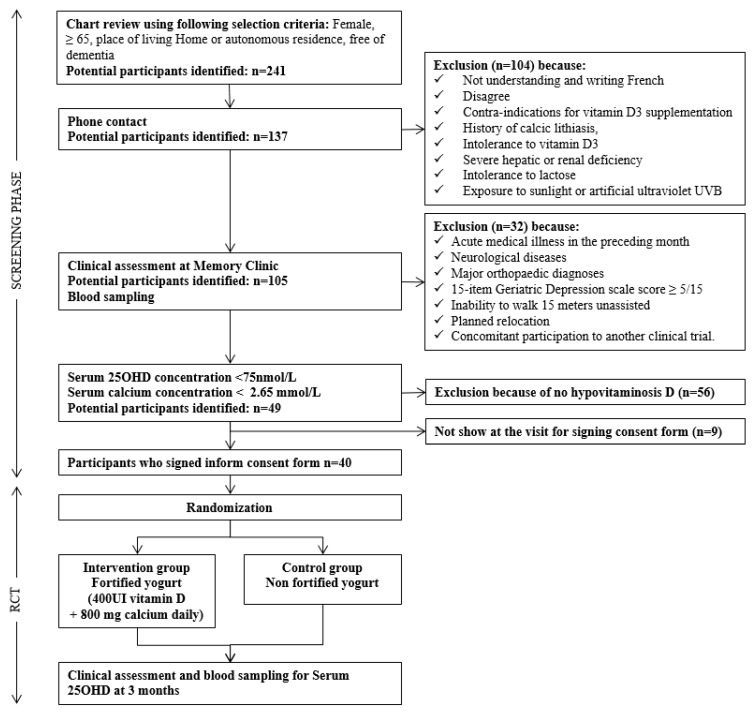
Flow chart of the selection of participants. RCT: Randomized controlled trial; 25OHD: 25-hydroxyvitamin D; UVB: ultraviolet B.

A flow chart of the consecutive steps of recruitment is shown in Figure 1. First, potential participants were screened through a chart review of individuals who were evaluated at the Memory Clinic of Angers University Hospital between June 2013 and May 2014. Secondly, the potential participants (*n* = 241) who were females, aged 65 and over, free of dementia and living at home or in an autonomous residence were contacted by phone. Thirdly, after applying the selection criteria (Figure 1), a total of 137 (56.9%) individuals came to the Memory Clinic of Angers University Hospital for a clinical assessment and a blood sampling with the objective to determine their serum 25OHD and calcium concentrations. 32 in these individuals were excluded due to the clinical exclusion criteria. Fourthly, the 105 (43.6%) remaining potential participants had a blood sampling. If they presented with hypovitaminosis D (serum 25OHD concentration < 75 nmol/L) and serum calcium level < 2.65 mmol/L, they came back to the Memory Clinic to sign the consent form. Lastly, 40 (29.2%) participants met all the selection criteria and were enrolled in the study. There were four waves of recruitment from September 2014 to January 2015. The last follow-up for the last recruited participant was April 2015.

### 2.2. Study Design 

The GAit, MEmory, Dietary and vitamin D (GAME-D2) is a unicentre (Memory clinic of Angers University Hospital, Angers, France), single-blind (principal investigator and support staff blinded), randomized, controlled, superiority clinical trial in two parallel groups (20 participants in the intervention group and 20 in the control group) with intent-to-treat. This RCT was registered on the ClinicalTrials.gov website (project number NCT02086409) and respects the CONSORT guidelines for RCTs [21].

All the recruited participants were randomized into either the intervention or the control group. Participants were randomly allocated with a 1:1 *ratio* to fortified yogurt and control (i.e., non-fortified) yogurt. Four randomization lists following the four waves of recruitment were established by an independent pharmacist of the Clinical Research Centre of Angers University Hospital with the use of the N’Query randomization software. In order to limit potential imbalances, a block randomization was used. Randomization lists were transmitted to the central pharmacy of Angers University Hospital, which was in charge of assigning the participants’ yogurt (fortified yogurt *versus* control) in compliance with the randomization list. The principal investigator and staff involved in the recruitment and follow-up of participants were "blinded", except one staff member who was neither involved in the recruitment nor the evaluation (at baseline/follow-up) of participants.

### 2.3. Intervention Versus Control Groups

The intervention was the dietary intake of fortified yogurt. Participants in the intervention group received at home, through a certified delivery company under the supervision of the central pharmacy of Angers University Hospital, fortified yogurt every two weeks during three consecutive months. The fortified yogurt contained proteins, carbohydrates and lipids with an additional 200 IU of vitamin D_3_ and 400 mg calcium per yogurt pot. Participants in the intervention group consumed two fortified yogurt pots per day during three consecutive months. The same procedure and rules listed above were followed for participants in the control group to receive the control yogurt pots at home. The control yogurt pots contained similar proteins, carbohydrates and lipids, along with no vitamin D_3_ supplementation and a lower dose of calcium per pot (150 mg). Both the fortified and control yogurts were presented in the form of a yogurt pot of 125 g and were packed in sets of 4 pots of 125 g. The yogurts used in the intervention and the control groups was marketed yogurt available to the general public. It was not possible to manufacture yogurt separately for this RCT because of the high costs for a unicentre trial.

### 2.4. Baseline and Follow-Up Assessment

The baseline assessment (M0) and follow-up assessment at three months (M3) were similar and included a full-standardized examination, physical tests and questionnaires. Recorded clinical characteristics were age, anthropometric measures (i.e., weight and height) with the calculation of body mass index (BMI, [Weight (kg)/Height (m)^2^], use of psychoactive drugs (i.e., antidepressants, benzodiazepines, hypnotics or neuroleptics), binocular visual acuity at a distance of 5m with the Snellen letter test chart score ranging from 0 (i.e., worst performance) to 10 (i.e., best performance) [22], lower-limb proprioception evaluated with a graduated tuning fork placed on the tibial tuberosity measuring vibration score ranging between 0 (i.e., worst performance) to 8 (i.e., best performance); the mean value obtained for the left and right sides was used in the study. Engaging in regular physical activity was assessed with the question: “Have you been engaged in one of the following recreational physical activity (walking, gymnastics, biking, swimming or gardening) for at least one hour a week in the past month or more?” In addition, the Cumulative Illness Rating scale geriatric form and the Mini Nutritional Assessment short form were completed [23].

Spatio-temporal gait parameters, including stride time, swing time and stride width, were recorded at a self-selected usual pace using a computerized walkway with embedded pressure sensors (GAITRite^®^ Gold walkway, 972 cm long, active electronic surface area 792 × 610 cm, total 29,952 pressure sensors, scanning frequency 60 Hz, CIR System, Havertown, PA, USA), according to the European guidelines for spatio-temporal gait analysis in older adults [24]. Briefly, the participants were asked to walk at their usual self-selected walking speed in a quiet well-lit corridor wearing their own footwear. To avoid acceleration and deceleration effects, participants started walking one meter before reaching the electronic walkway and completed their walk one meter beyond it. For each parameter, mean value and coefficient of variation (CoV = (standard deviation/mean) × 100) were recorded. The maximal isometric voluntary contraction (MVC) strength of hand was measured with computerized hydraulic dynamometers (Martin Vigorimeter, Medizin Tecnik, Tutlingen, Germany). The test was performed three times with the dominant arm. Finally, a face-to-face neuropsychological assessment was performed by a neuropsychologist. The level of education of participants was assessed with the number of school years and high education levels were determined using a threshold ≥ 10. Mood was assessed with the 15-item GDS with a score ranged from 0 (i.e., absence of active depressive symptomatology) to 15 (i.e., severe depressive symptomatology) [20]. Several aspects of cognitive function were examined and included Mini Mental State Examination (MMSE) [25], Frontal Assessment Battery (FAB) [26], Trail Making Test (TMT) parts A and B [27], direct and indirect digit span score [28], and Stroop test [29].

Blood samples (from a fasting blood test) were obtained by a trained nurse at the end of the screening phase/baseline assessment (M0) and at 3 months (M3) for measurement of serum 25OHD concentration. All the measurements were performed at Angers University Hospital, France. Serum 25OHD concentration was measured by radioimmunoassay (DiaSorin, IncstarCorp, Stillwater, MN, USA). With this method, there is no lipid interference, which is often observed in other non-chromatographic assays of 25OHD. The intra- and inter-assay precisions were 5.2% and 11.3%, respectively [5].

Compliance was assessed by having study participants collect and return the lids of the fortified and the control yogurt pots to the principal investigator or staff member at the last follow-up visit (i.e., at 3 months). It was assumed that the participant was compliant if they brought back at least 80% of the yogurt pot lids at the M3 visit.

### 2.5. Outcomes

The primary outcome was the CoV of stride time at the baseline assessment (M0) and at the end of the follow-up (M3). It was assessed using a standardized procedure and with a computerized walkway with embedded pressure sensors, as described above [30]. The secondary outcomes were the following values at the baseline assessment (M0) and at the 3-month follow-up (M3):

Mean value of stride time; mean values and CoV of stride velocity, swing time and stride width.

Mean value of handgrip strength.

Mean value of serum 25OHD concentration.

Mean value of MMSE and FAB score; time to perform TMTA and TMTB, and *ratio* of TMT (i.e., TMTB/TMTA); numbers of direct and indirect digit recalls in correct order; time to perform Stroop color test Part I, Stroop word Part II, Stroop color-word test Part III, and a *ratio* score of Stroop (i.e., (‘‘No Interference” [Color])/‘‘Interference” [color-word]).

Variation, calculated with the formula ([value M0–value M3]/((value M0–value M3/2)) × 100, of values of spatiotemporal gait parameters (mean value and coefficient of variation), MMSE, FAB, TMTA and TMTB, direct and indirect digit span score, and Stroop scores; handgrip strength; serum 25OHD concentration.

Clinical adverse effects measured by phone contact in case of occurrence (patients called the principal investigator or staff member to inform them about the occurrence of an adverse effect and to make a decision of whether to withdraw from the study) and at the end of the follow-up period.

Compliance, checked by collecting the yogurt pot lids kept by the participants and brought back to the principal investigator or support staff at the last follow-up visit (i.e., M3).

### 2.6. Standard Protocol Approvals, Registrations, and Patient Consents

This study was conducted in accordance with the ethical standards set forth in the Helsinki Declaration (1983). The participants in the study were included after obtaining written informed consent for the project. The local ethics committee of Angers University Hospital approved the project.

### 2.7. Statistics

The sample size was determined using the primary outcome (i.e., CoV of stride) at the baseline visit and at the last follow-up visit. It was considered that, in an RCT with comparable groups of healthy older individuals, the mean value of CoV was 2.5% [6]. To detect a clinically significant change of 1% of CoV, a total of 20 individuals per group were needed, assuming a power of 90% and a two-sided alpha of 5%. The normal range of CoV of stride time is limited and between 2% to 3% in healthy older adults [6,31]. Thus, a gross change of 1% of stride time CoV means a relative change around one third. All statistical analyses were performed with the intention to treat for all enrolled participants randomized in both groups (i.e., intervention *versus* control).

The participants’ characteristics were summarized using means and standard deviations (SD) or frequencies and percentages, as appropriate. Participants were separated into 2 groups: intervention *versus* control. Inter-group comparisons were performed using the Mann–Whitney U test or Chi-square test, as appropriate, whereas, the Wilcoxon or Chi-square tests were used for intra-group comparisons. The changes in outcomes at the end of the follow-up (M3) were significantly different between the intervention and the control groups, when compared using Mann–Whitney U test. The changes were expressed in percentage and calculated from the formula: ([Value at baseline assessment-value at end of follow-up period]/([value at baseline assessment-value at end of follow-up period]/2)) × 100. In addition, to characterize and compare the magnitude of changes, the ’mean difference’ of each significant outcome between participants in the intervention and the control groups was graphed. The mean difference is commonly used for meta-analysis of continuous data. The mean difference data were analyzed with a fixed-effects meta-analysis strategy, generating a summary measure of the mean difference [95% confidence interval (CI)] of each parameter of the intervention and the control participants. The results are presented as forest plots. Heterogeneity between outcomes was assessed using the Cochran’s Chi-squared test for homogeneity (Chi^2^) and the amount of variation due to heterogeneity was estimated by calculating the I^2^. Statistical analyses were performed using the software programs Review Manager (RevMan) version 5.1 (The Nordic Cochrane Centre, Copenhagen, Denmark) and SPSS (version 23.0; SPSS, Inc., Chicago, IL). For comparisons of baseline characteristics of participants between the intervention and the control groups, the *p*-values were considered significant if <0.0013 because of multiple comparisons (*n* = 37). For all the other statistical tests, *P*-values less than 0.05 were considered statistically significant.

## 3. Results

The nutrition values of the fortified yogurt are presented in Table 1. The fortified yogurt contained 200 UI of vitamin D_3_ and 400 mg calcium, whereas the non-fortified yogurt had a lower dose of calcium (150 mg) and no vitamin D_3_ supplementation. Both fortified and non-fortified yogurts contained similar proteins, carbohydrates and lipids. The compliance was good as all participants returned more than 80% of the yogurt pot lids at the end of the follow-up (i.e., at 3 months). A limited number of participants dropped out during the study (*n* = 3, 7.5%). One participant (2.5%) in the intervention group withdrew her consent, because of minor and transitory digestive disorders (i.e., nausea) and two participants (5%) in the control group withdrew because of minor and transitory digestive disorders (i.e., nausea), as well as a coagulation event (i.e., overdose). Table 2 presents the baseline characteristics of the intervention and the control groups. There was no significant difference between groups at the baseline assessment, regardless of the characteristics compared. Inter-group comparisons at the end of the follow-up showed that stride time and swing time variability were lower (i.e., greater performances) in the intervention group compared to the control group (1.9 ± 0.5% *versus* 2.7 ± 1.6% with *p* = 0.049, and 2.9 ± 0.7% *versus* 4.5 ± 2.3% with *p* = 0.002). In addition, the MMSE score was higher (i.e., greater performance) in the intervention group compared to the control group (28.5 ± 0.9 *versus* 27.1 ± 1.9 with *p* = 0.010) at the end of the follow-up. The intra-group comparison showed that time to perform the TMTB was longer (i.e. worse performance) at the baseline assessment compared to the end of the follow-up in the intervention group (106.0 ± 32.1 *versus* 88.8 ± 25.2 with *p* = 0.035). The Stroop color-word part III was worse at the baseline assessment compared to the end of the follow-up in the control group (134.2 ± 24.5 *versus* 126.3 ± 29.3 with *p* = 0.047).

**Figure 2:**
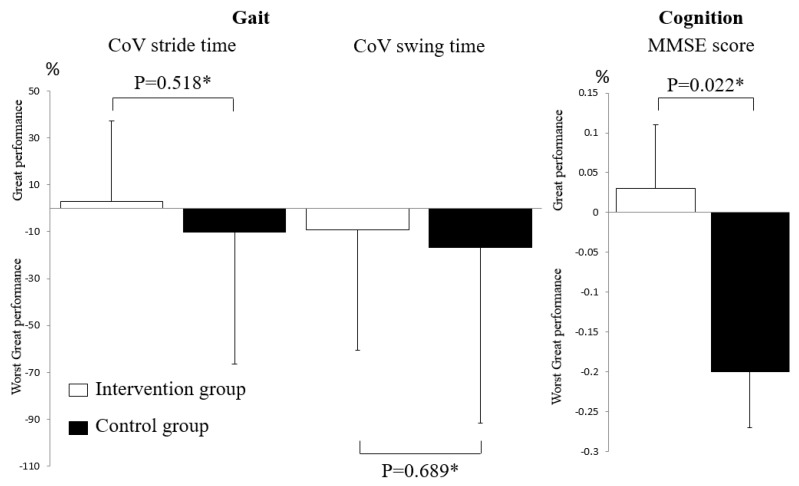
Changes in gait and cognitive performances from baseline assessment to the end of the follow-up period in participants randomized in intervention (*n* = 20) and placebo groups (*n* = 20). CoV: Coefficient of Variation; MMSE: Mini Mental State Examination; only gait and cognitive variables significantly (*p* < 0.05) different between the intervention and the control group at the end of follow-up period were studied; *: Comparison based on Mann–Whitney test.

**Figure 3:**
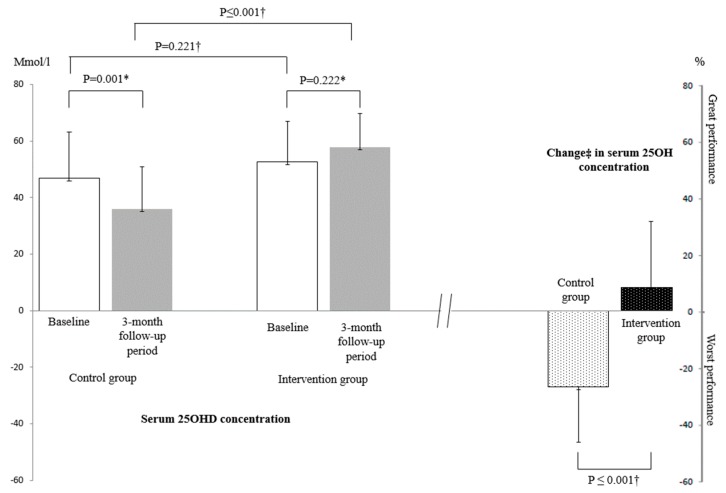
Serum 25-hydroxyvitamin D concentrations and changes from baseline assessment to end of follow-up period in participants randomized in intervention (*n* = 20) and control groups (*n* = 20). 25OHD: 25-hydroxyvitamin D. *: comparison based on Wilcoxon test. †: comparison based on Mann-Whitney test. ‡: calculated from the formula: ([25-hydroxyvitamin D at baseline assessment - 25-hydroxyvitamin D at 3-month follow-up period]/([25-hydroxyvitamin D at baseline assessment -25-hydroxyvitamin D at 3-month follow-up period]/2)) × 100.

As shown in Figure 2, inter-group comparisons of changes in CoV of stride time and swing time between the baseline and end of the follow-up assessments did not differ significantly (*p* = 0.518 and *p* = 0.689), whereas the change in MMSE was significant (*p* = 0.022), with a higher score being shown in the intervention group compared to the control group. Serum 25OHD concentrations did not differ between the intervention and the control groups at the baseline (*p* = 0.221) assessment, but were different at the end of the follow-up, with higher serum concentrations being shown in the intervention compared to the control groups (*p* ≤ 0.001). In addition, serum 25OHD concentrations increased in the intervention group between the baseline and the end of the follow-up assessments, but this increase was not significant (*p* = 0.222); comparatively, it decreased significantly in the control group (*p* = 0.001). These changes in serum 25OHD concentrations were significant (*p* < 0.001, Figure 3).

**Figure 4:**
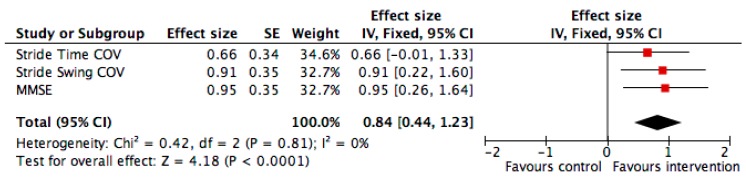
Effect size* of significant effects in the intervention group compared to the control group for gait parameters and cognition (i.e., coefficient of variation of stride time and swing time) and cognition (i.e., mini mental state examination score) (*n* = 40). *: Effect size was calculated from the mean value and standard deviation of Coefficient of Variation of stride time and swing time and on the mean Mini Mental State Examination score in participants in the intervention and control groups.

Finally, the effect size of significant changes in the intervention group compared to the control group demonstrated a significant change for the CoV of stride swing and the MMSE score, with an overall significant improvement when combining all changes of outcomes (*p* ≤ 0.001, Figure 4).

Change expressed in percentage and calculated from the formula: ([Value at baseline assessment-value at the end of the follow-up period]/([value at baseline assessment-value at end of follow-up period]/2)) × 100, only parameters significantly different between intervention and control groups at the end of the follow-up period (i.e., Coefficient of Variation of stride time and swing time, and Mini Mental State Examination score) used in this analysis.

An effect size calculator worksheet was used to derive effect size and standard error from mean and standard deviations and the size of each group (Coe’s Calculator retrieved 25 March 2015 from http://www.cemcentre.org/evidence-basededucation/effect-size-calculator). CoV: Coefficient of Variation.

The red square area is proportional to the number of individuals in each group and the horizontal lines correspond to the 95% confidence interval. The vertical line corresponds to no difference compared to the individuals in the control group. A greater performance corresponds to an improvement of the parameters in the intervention group compared to the control group.

## 4. Discussion

The findings show mixed results for spatiotemporal gait parameters. There was no significant change between the intervention group compared to the control group from the baseline to the end of the follow-up; however, the CoV of stride time and swing time were lower (i.e., greater performance) in the intervention group compared to the control group at the end of the follow-up. In contrast, global cognitive performance and serum 25OHD concentrations were stable in the intervention group, whereas a decrease of these parameters was observed in the control group; these changes were significantly different between groups, with higher values (i.e., greater performance) reported in the intervention group compared to the control group.

The first main result of the RCT is the mixed effects of fortified yogurt on spatio-temporal gait parameters. No significant change was reported; however, cross-sectional comparisons at the end of the follow-up showed lower gait variability in the intervention group compared to the control group. In terms of control of gait, gait variability has been identified as an appropriate biomarker of efficient gait control [31]. The general assumption is that variability and stability are negatively correlated, lower variability reflecting better gait stability, performance and control [31]. The range of variability was low and normal in participants (i.e., 1.9%–2.7% in both groups), underscoring their relatively good health condition. For instance, it has been shown that in healthy older adults, stride time variability is below 3%, which was the case for our cohort [6,31]. Even though no significant change between the baseline and the end of the follow-up assessment of gait variability were reported, the novelty of GAME-D2 RCT is to show that it is possible to prevent increased gait variability in the intervention group compared to the control group. At the end of the 3-month period of the intervention, there was a significant difference between groups, with the lowest gait variability being reported in the intervention group for the stride time variability. This gait variability may be related to the stabilization of the mean value of serum 25OHD concentration and the significant change of vitamin D status in the intervention group compared to the control group. Indeed, after 3 months of consuming fortified yogurt daily, a majority of females in the intervention group had insufficient serum 25OHD levels, whereas those in the control group were deficient. In addition, our study confirms that the gait variability, and in particular, stride time variability is a temporal gait parameter associated with serum 25OHD concentration. Indeed, we previously showed that greater stride time variability (i.e., worst performance) was associated with lower serum 25OHD concentrations [6].

The second main finding of the GAME-D2 RCT is that the cognitive performance improved in the intervention group compared to the control group after 3 months of fortified food supplementation in older adults. This better performance in the intervention group was limited to MMSE score, which assesses global cognitive functioning. This result is in concordance with previous studies, which reported positive association between serum 25OHD and cognitive performance [5]. Furthermore, it has been shown in an open label trial that vitamin D supplementation using 100,000 IU per month for one year improved the MMSE score in older adults free of dementia with hypovitaminosis D [30]. However, although it has been reported that executive functions were especially related to serum 25OHD concentrations in individuals free of dementia and that supplementation may also improve executive performance, no association was shown with this subdomain of cognitive function in this RCT [5,32].

This RCT did not show any improvement in muscle strength in the intervention group. This result is consistent with the literature that reports mixed results, as some studies showed positive effects and others observed non-conclusive effects [33,34]. One explanation for the absence of improvement of muscle strength with vitamin D in our study may be related to the low level of supplementation, which was limited to 400 IU per day. Indeed, positive effects on muscle have usually been demonstrated with higher doses of vitamin D supplementation [34]. In addition, the relatively good health condition of the participants, who were well-functioning females may also be a limitation to show an effect on muscle strength in our RCT.

All the participants of the GAME-D2 RCT had hypovitaminosis D defined by serum 25OHD concentration < 75 nmol/L. Among them, around 50% had concentrations < 50 nmol/L at baseline defining vitamin D deficiency regardless of the group considered (i.e., intervention *versus* control). In contrast, at the end of the follow-up, only 20% were deficient in the intervention group and 65% in the control group. Thus, following consumption of the vitamin D fortified yogurt, a majority of females in the intervention group had a vitamin D insufficiency and two had no hypovitaminosis D, whereas, all the control participants still had hypovitaminosis D and most of them were deficient. This opposite change in serum 25OHD concentrations (i.e., improvement of vitamin D status in the intervention group versus worsening in the control group) was associated with greater extra-skeletal performances in the intervention group compared to the control group. This result supports the 2011 report of the Institute of Medicine, which estimated that serum 25OHD concentrations ≥ 50 nmol/L meets the needs, primarily related to bone health outcomes [35]. This RCT highlights that this threshold also seems to prevent the risks of adverse extraskeletal outcomes. Two previous RCTs, using the same fortified yogurt taken by the intervention group, reported a significant increase in serum 25OHD concentration [14,15]. Compared to the other study, we did not report a significant increase but a stabilization of mean serum 25OHD concentrations. However, we observed a significant change in the vitamin D status in the intervention group because after three months, a majority of them were insufficient and deficient, which was not the case in the control group due to a seasonality effect. Indeed, in the control group, there was a significant decrease in serum 25OHD concentrations and most of the females in this group were deficient at the end of the study. This result is, therefore, in concordance with the two previous RCT [14,15].

All the females of GAME-D2 RCT were compliant with the fortified yogurt. This high compliance of our participants is an argument to suggest that the compliance of supplemental vitamin D as provided through the regular consumption of fortified dairy products should be beneficial. In terms of public health, it has been reported that compared to routine supplementation in a healthy population to achieve adequate vitamin D status, the preferable option is to encourage the public to choose foods containing or fortified with vitamin D [35].

The GAME-D2 study has a number of strengths, including the design, which was an RCT, with well-balanced characteristics of both groups at the baseline, targeting the extra-skeletal outcomes, examining healthy females and a potential utility in terms of strategy to maintain a normal vitamin D status through fortified food. However, some limitations must be considered. First, we did not use a *placebo* yogurt but a control yogurt and the RCT was a single blind trial. Participants knew whether they were in the intervention or the control group, which may influence the effects on the examined outcomes. Secondly, there were no males recruited in the RCT. Sex (i.e., female versus male) may influence the effects of the GAME-D2 outcomes due to the various biological characteristics specific to females. Thirdly, the number of participants was low (*n* = 40) and all of them were recruited from one French centre, which was a Memory clinic where they consulted for memory complaints, limiting the generalizability of our results. Fourthly, even though there was no significant difference between the groups for spatiotemporal gait characteristics, the stride velocity was higher in the intervention group compared to the control group (around 120 cm/second *versus* 110 cm/second) and this difference of 10 cm/second may have had an effect on other gait parameters [36].

## 5. Conclusions

In conclusion, fortified yogurt with vitamin D (i.e., 200 IU) and calcium (i.e., 400 mg) twice a day maintain cognitive performances and vitamin D status in older females but not gait performances, suggesting that they may prevent hypovitaminosis D-related extra-skeletal disorders.

**Highlights**

− Hypovitaminosis D is associated with skeletal and extra-skeletal disorders.
− Vitamin D_3_ and calcium fortified food may improve serum vitamin D status.
− Fortified yogurt with vitamin D had no effect on gait performances.
− Fortified yogurt with vitamin D and calcium maintained global cognitive performance and serum vitamin D status.
− The greatest improvement reported with fortified yogurt was observed with cognitive performance.

## Figures and Tables

**Table 1:** Nutrients characteristics of fortified and control yogurts.

	Per 125 g Pot	
	Fortified Yogurt	Control Yogurt
Energy	377 kJ (90 kcal)	370 kJ (90 kcal)
Protein	5.0 g	5.0 g
Carbohydrate	5.7 g	7.0 g
Of which sugar	5.3 g	7.0 g
Fat	4.3 g	4.5 g
Of which saturated	2.8 g	3.0 g
Sodium	0.08 g	0.075 g
Calcium	400 mg	150 mg
Vitamin D	200 IU	-

kJ: kilo Joules; kcal: kilo calories.

**Table 2:** Baseline characteristics of participants randomized in intervention (*n* = 20) and control groups (*n* = 20).

	Participants			*P*-Value *
	Total (*n* = 40)	Intervention Group (*n* = 20)	Control Group (*n* = 20)	
Age (years), mean ± SD	71.2 ± 4.4	71.0 ± 3.7	71.5 ± 5.2	0.947
CIRS-G score (/70), mean ± SD	4.9 ± 2.9	5.0 ± 3.5	4.9 ± 2.4	0.620
Use of psychoactive drugs †, *n* (%)	8 (20.0)	3 (15.0)	5 (25.0)	0.429
Body mass index (kg/m^2^)				
Mean ± SD	27.0 ± 3.7	27.6 ± 4.1	26.3 ± 3.3	0.414
<21, *n* (%)	1 (2.5)	0 (0)	1 (5.0)	0.311
MNA-SF score				
Mean ± SD (/14)	13.3 ± 1.2	13.3 ± 1.4	13.4 ± 1.1	0.947
Under-nutrition (score < 12), *n* (%)	5 (12.5)	3 (15.0)	2 (10.0)	0.633
Regular physical activity ‡, *n* (%)	36 (90.0)	18 (90.0)	18 (90.0)	1.000
Usual walking speed # (m/s), mean ± SD	114.9 ± 20.0	120.5 ± 17.9	109.4 ± 20.8	0.091
Distance visual acuity || (/10), mean ± SD	8.2 ± 1.6	8.3 ± 1.7	8.0 ± 1.5	0.414
Lower limb proprioception ¶ (/8), mean ± SD	6.0 ± 1.1	6.1 ± 0.9	5.8 ± 1.4	0.301
Handgrip strength § (N), mean ± SD	26.1 ± 2.7	26.3 ± 2.6	26.0 ± 2.8	0.369
15-item GDS score				
Mean ± SD (/15)	1.9 ± 1.6	1.8 ± 1.8	2.1 ± 1.5	0.461
Score > 5, *n* (%)	5 (12.5)	4 (20.0)	1 (5.0)	0.151
High educational level **, *n* (%)	24 (60.0)	13 (65.0)	11 (55.0)	0.519
MMSE score (/30), mean ± SD	27.7 ± 2.0	27.8 ± 2.3	27.7 ± 1.8	0.602
FAB score (/18), mean ± SD	16.1 ± 1.6	16.4 ± 1.6	15.9 ± 1.7	0.201
Serum 25OHD concentration (nmol/L)				
Mean ± SD	49.8 ± 15.4	52.6 ± 14.3	47.0 ± 16.2	0.221
<50, *n* (%)	19 (47.5)	9 (45.0)	10 (50.0)	0.752
Gait characteristics ††				
Stride velocity				
Mean ± SD (cm.s^-1^)	115.8 ± 20.2	121.4 ± 18.1	110.1 ± 21.0	0.076
CoV (%)	4.2 ± 1.7	4.0 ± 1.8	4.4 ± 1.6	0.301
Stride time				
Mean ± SD (ms)	1079.0 ± 111.5	1041.9 ± 74.6	1116.1 ± 130.5	0.192
CoV (%)	2.2 ± 0.9	2.0 ± 0.9	2.3 ± 0.9	0.183
Swing time				
Mean ± SD (ms)	387.4 ± 32.0	376.2 ± 24.1	398.7 ± 35.4	0.033
CoV (%)	6.7 ± 2.4	2.9 ± 1.2	3.9 ± 1.8	0.081
Stride width				
Mean ± SD (cm)	6.8 ± 2.4	6.7 ± 2.0	6.8 ± 2.8	0.799
CoV (%)	35.2 ± 16.4	31.4 ± 13.6	39.0 ± 18.3	0.183
Handgrip strength ‡‡ (N.m^-2^), mean ± SD	26.1 ± 2.7	26.0 ± 2.9	26.1 ± 2.6	0.883
Cognitive performance, mean ± SD				
MMSE score (/30)	27.7 ± 2.0	27.8 ± 2.3	27.7 ± 1.8	0.602
FAB score (/18)	16.1 ± 1.6	16.4 ± 1.6	15.9 ± 1.7	0.201
TMTA ## (s)	46.7 ± 13.6	47.9 ± 12.4	45.5 ± 15.0	0.341
TMTB ## (s)	114.7 ± 35.0	106.0 ± 32.1	124.5 ± 36.5	0.186
Direct Digit Span score ||||	5.6 ± 1.1	5.5 ± 1.1	5.8 ± 1.1	0.529
Indirect Digit Span score ¶¶	4.1 ± 1.1	4.1 ± 1.0	4.2 ± 1.2	0.547
Stroop Color test Part I §§	65.4 ± 12.9	61.2 ± 12.0	69.4 ± 12.8	0.079
Stroop Word test Part II ***	45.8 ± 7.2	45.3 ± 7.5	46.2 ± 7.0	0.923
Stroop Color-Word test Part III†††	132.2 ± 30.3	130.3 ± 35.7	134.2 ± 24.5	0.563

SD: Standard deviation; CIRS-G: Cumulative Illness Rating Scale Geriatric form; MNA-SF: Mini Nutritional Assessment Short Form; GSD: Geriatric Depression Scale; MMSE: Mini Mental State Examination; FAB: Frontal Assessment Battery; FAB: Frontal Assessment Battery; TMT: Trail Making Test; N.m^-2^: Newton per square meter; 25OHD: 25-hydroxyvitamin D; s: Second; ms: Millisecond; N.m^-2^: Newton per square meter; *: Comparison based on Mann-Whitney U test or Chi-square test, as appropriate; †: Use of benzodiazepines or antidepressants or neuroleptics; ‡: At least one recreational physical activity (walking, gymnastics, cycling, swimming or gardening) for at least one hour a week for the past month or more; #: Measured with GAITRite® system at steady-state walking; ||: Binocular visual acuity at a distance of 5m with a Snellen letter test chart; ¶: Mean value of left and right sides, based on graduated tuning fork placed on the lower limb; §: Mean value of the three trials measuring the highest value of maximal isometric voluntary contraction strength recorded with computerized dynamometers; **: Number of school years > 10; ††: Gait analysis performed with GAITRite® system; ‡‡: Maximal isometric voluntary contraction strength recorded with computerized dynamometers, mean value of 3 trials; ##: Times to “connect-the-dots” as quickly as possible of 25 consecutive targets on a sheet of paper; in part A the targets are numbers and in part B alternated numbers and letters; ||||: Total number of digits that an individual can absorb and recall in correct forward serial orders after hearing them; ¶¶: Total number of digits that an individual can absorb and recall in correct backward serial orders after hearing them; §§: Time to name color; ***: Time to read word; †††: Time to name color of word, *p*-value significant (i.e., <0.0013 because of multiple comparisons *n* = 37) indicated in bold.
